# Mechanisms for reducing/eliminating chronic neuropathic pain with a focus on platelet-rich plasma

**DOI:** 10.3389/ebm.2025.10567

**Published:** 2025-06-30

**Authors:** Damien P. Kuffler, Christian A. Foy

**Affiliations:** ^1^ Institute of Neurobiology, Medical School, University of Puerto Rico, San Juan, PR, United States; ^2^ Department of Orthopedic Surgery, Medical School, University of Puerto Rico, San Juan, PR, United States

**Keywords:** axon regeneration, chronic neuropathic pain, nerve repair, pain elimination, plateletrich plasma, platelets

## Abstract

Peripheral nerve trauma commonly results in chronic neuropathic pain by up-regulating the synthesis and release of pro-inflammatory mediators from local and invading cells and inducing hyperexcitability of nociceptive neurons and spontaneous electrical activity. The pain decreases when these cells down-regulate genes supporting the pro-inflammatory state, up-regulate genes for expressing anti-inflammatory factors, and modulate genes that reduce nociceptive neuron spontaneous electrical activity. Pharmacological agents, the primary technique for reducing pain, do not eliminate pain, and <50% of patients achieve benefits because they do not address the underlying causes of pain. Alternative techniques providing longer lasting, but not complete or long-term pain relief include surgical interventions, electrical stimulation, and antibody treatment. Anti-inflammatory mediators can reduce pain, but the effect is not complete or long-lasting. Platelet-rich plasma (PRP) contains a readably available evolutionarily developed cocktail of factors that induce longer-lasting and more significant, but not complete, pain relief than other techniques. However, a novel study shows that unique formulations of PRP can induce long-term pain elimination. This review examines (1) the efficacy of drugs, regenerative peripheral nerve interface (RPNI), targeted muscle reinnervation (TMR), and PRP in reducing chronic neuropathic pain, (2) recent clinical data showing that a novel PRP application technique induces long-term chronic neuropathic pain reduction/elimination, and (3) discusses why the novel PRP may be more effective in reducing/eliminating chronic neuropathic pain.

## Impact statement

Peripheral nerve trauma and surgical interventions result in 60% of individuals suffering chronic neuropathic pain. The standard technique for reducing pain is pharmacological agents, although they may not be effective, may reduce but not eliminate pain, are not long-lasting, are strongly addictive, and their side effects may preclude their use. Physiologically, chronic pain is reduced/eliminated when injured axons reinnervate their targets. However, because, following nerve repairs, <50% of patients recover function, most patients suffer chronic pain. Novel techniques are required that induce meaningful recovery or directly reduce/eliminate chronic neuropathic pain. This review examines the efficacy of pharmacological agents and other techniques for their analgesic efficacies. It then discusses a novel technique involving platelet-rich plasma (PRP), which reliably and rapidly induces long-term chronic neuropathic pain reduction/elimination. Finally, it briefly discusses various platelet-released factors that may be responsible for this influence and their mechanisms of action.

## Introduction

Up to 16% of the US population suffers chronic neuropathic pain due to trauma, amputation, and surgery [1, 2], with peripheral nerve trauma and surgical interventions leading to pain in 60% of patients [3–5]. For those who undergo peripheral nerve surgical procedures, one study found >50% have significant pain reduction [6], while another 73% continued to have or developed pain [7]. The pain was chronic and intense for about 30% [3, 8], debilitating for many [9], and challenging to treat [10, 11]. Of patients presenting to pain clinics reporting chronic neuropathic pain, 78% suffered pain after 6 months, decreasing to 56% after 12 months [12].

Surgical interventions [13–15], electrical stimulation [16–18] antibodies against pro-inflammatory mediators and their receptors [19, 20], and drugs that block nociceptive neurons’ hyperexcitability and spontaneous ectopic electrical activity [21–23] provide long-term chronic neuropathic pain relief, but not elimination, to <50% of patients [24, 25].

Extensive evidence shows that injury-induced inflammation underlies neuropathic pain [3]. This suggests that administering anti-inflammatory agents should reduce chronic pain. However, clinically, administering anti-inflammatory drugs prolongs rather than shortens the time to pain elimination, while administering pro-inflammatory mediators reduces pain more rapidly [26]. While counterintuitive, this is because inflammation induces neutrophil invasion and up-regulates the synthesis and release of pro-inflammatory factors, which trigger an anti-inflammatory response [26]. Therefore, reducing/eliminating chronic neuropathic pain requires understanding which cells are recruited by injuries, the sequences of their recruitment, and what leads to the up- or down-regulation of specifically released factors.

This paper examines the efficacy of drugs and PRP in reducing pain and the results of two novel clinical techniques involving PRP, which induce long-term chronic neuropathic pain reduction/elimination. Finally, the paper discusses the pro-algesic and analgesic roles played by some platelet-released factors that induce pain reduction/elimination.

### Pharmaceutical agents

Clinically, pharmacological agents are best for reducing pain and providing adequate pain control to 30%–40% of patients [27]. Among the most effective opioid receptor agonists are strong [1, 28–34] followed by weak [35, 36] opioids [28], anticonvulsive drugs [37], such as gabapentin [38–42], tricyclic antidepressants [43], and the selective norepinephrine and anti-epileptic drug pregabalin [44]. While opioids are the most effective [45], their efficacies are increased by combining them with other drugs [46]. The clinical efficacies of other techniques, such as the local application of capsaicin [47]and lidocaine [48], are less well-established and are still being tested [49]. Recently, suzetrigine was FDA approved (first in class JAN 2025) as a non-opioid analgesic of comparable efficacy to higher-potency opioids. [50]. Its efficacy compared to PRP is not known. However, it has been shown to induce mild to moderate severe adverse events [50], while PRP induces no known adverse events.

The anesthetic ketamine is effective against chronic neuropathic pain [51]. It is considered to act by inhibiting the N-methyl-D-aspartate (NMDA) receptor and possibly other mechanisms, such as enhancing descending inhibition and central site anti-inflammatory actions [51]. However, short-term NMDA infusions induced potent analgesia only during its administration, while prolonged infusion (4–14 days) induces analgesia for up to 3 months following infusion [51]. Unfortunately, ketamine’s clinical side effects include nausea/vomiting, psychedelic symptoms (hallucinations, memory defects, panic attacks), cardiovascular stimulation, and somnolence, with a minority of patients suffering hepatotoxicity [51].

No pharmacological agent provides long-term analgesia [52], and for patients with chronic pain, 54% use opioid medications daily, despite up to 97% reporting inadequate pain relief [6, 53]. However, their use is limited because of adverse effects [54, 55] and problems with abuse, dependence, overdose, and death [54, 55]. Therefore, it is essential to balance opioid pain control and the development of opioid dependence [56]. These difficulties can be reduced by multimodal analgesic plans, non-opioid medications, and regional application techniques [31, 56]. Nevertheless, novel pain relief techniques are required [57], including developing alternative forms of nerve surgery [6], and pharmacological agents.

### Targeted muscle reinnervation (TMR) and regenerative peripheral nerve interface (RPNI)

Removing painful neuromas reduces but does not eliminate pain [13], and there is a high rate of neuroma and pain redevelopment [58]. However, following neuroma removal, the pain that normally develops is reduced by securing the nerve stump to an autograft or allograft [59, 60]. For lower extremity amputations, pain is reduced by nerve capping or implanting nerve stumps in bone [24, 61]. However, there is still no long-term chronic neuropathic pain reduction [62].

The most effective techniques for preventing or reducing chronic neuropathic pain or post-amputation neuroma pain are regenerative peripheral nerve interface (RPNI) and targeted muscle reinnervation (TMR) [63–67]. RPNI involves coapting a nerve stump into a small denervated muscle grafts, while TMR involves coapting the proximal nerve stump to the proximal motor nerve innervating a small muscle graft. Thus, following neuroma excision, both RPNI and TMR reduce pain development [68] and clinically reduce post-amputation neuroma pain in 75–100% of patients [64–67, 69–71] and phantom limb pain in 45–80%. However, TMR has the significant limitation of being only effective if applied <3 months post-trauma [72], requires sacrificing a motor nerve and cannot be used if a goal is to both reduce pain and restore function.

### Target reinnervation and cessation of axon regeneration

Abnormal spontaneous electrical activity of regenerating dorsal root axons is closely associated with chronic neuropathic pain [73–75]. Clinically, chronic pain reduction/elimination occurs only slightly before or in association with initial signs of functional recovery [76]. These findings led to the hypothesis that pain remains chronic when axons are regenerating [73] and only decreases or is eliminated when axons reinnervate targets, stop regenerating [63, 73, 77, 78], take up a target-derived factor/s [76, 79–81], which silence hyperexcitable nociceptive axons [63].

Supporting this hypothesis is that the extent of pain reduction decreases proportionately with the increasing extent of functional recovery [82, 83]. In rats, pain behavior is reduced or eliminated when axon regeneration is stopped/inhibited [73, 84], such as by applying semaphorin 3A [85] and injecting small-interfering RNA (siRNA) into axotomized sensory ganglia to block growth-associated protein-43 (GAP-43) expression [73, 86], This hypothesis is consistent with studies showing that TMR reduces/eliminates chronic neuropathic pain [69, 77, 87], including complex regional pain syndrome (CRPST) type II [88]. This idea is also consistent with rat chronic pain behavior being blocked by tetrodotoxin (TTX), GAP-43 knockdown, and semaphorin 3A, which stop axon regeneration and the electrical activity of nociceptive neurons [73]. Target reinnervation and the cessation of axon regeneration are consistent with TMR and RPNI reducing/eliminating chronic neuropathic pain, which occurs in 71%–100% of the subjects [66, 69].

### PRP and the reduction/elimination of chronic neuropathic pain

One of the challenges in using PRP is consistency in the findings between different studies. Thus, some clinical studies concluded that PRP provided little or no pain relief for tendinosis or rotator cuff tears [89–91]. Meta-analyses of multiple studies support this conclusion [89, 92, 93]. However, other clinical studies found that PRP reduced pain associated with tendinosis [94, 95], tendon injury [96–98], rotator cuff tears [99, 100] osteoarthritis [101, 102], plantar fasciitis [103], and muscle injuries [104]. These findings were supported by meta-analysis [105]. Animal model studies show PRP reduces pain caused by many types of injuries [106, 107], such as skin burn-induced neuropathic pain [108], painful lesions caused by *mycobacterium leprae* (leprosy bacteria) [109], and rat spinal cord injury sites [110]. Clinically, PRP also reduces peripheral nerve pain when applied to digital [111] and sciatic [112] nerve crush sites, pudendal nerve neurolysis surgery sites [113], the median nerve at the carpal tunnel’s proximal edge [114], and when injected into the perineurium of patients suffering from diabetic neuropathic pain [115]. These techniques result in >80% of patients achieving ca. three months of pain relief [116].

Clinically, a single epidural PRP injection provides lower back pain relief for up to 6 months [117, 118] while reducing complex chronic degenerative spinal pain [119]. Multiple PRP epidural injections provide temporary lumbar radicular pain relief [120], which is significantly increased by adding corticosteroids, although this is effective for only about 50% of patients [121, 122]. In comparative studies, the analgesic efficacy of PRP is similar to [120, 123] or better than [94, 124] that is provided by injecting a corticosteroid. An effective alternative technique for rats involves wrapping nerves in a PRP-coated NeuraWrap Nerve Protector [125]. Nevertheless, two meta-analyses found that in animal models and clinically, although PRP induces pain relief and nerve regeneration, the effect is not long-lasting [114, 126].

### Variability in PRP efficacy in reducing neuropathic pain

The efficacy of PRP in reducing pain and the duration of the suppression varies significantly between individuals [127] and studies [128]. This variability is best explained by significant differences in the composition of the PRP due to how it is prepared [129]. These techniques include single vs. double-spin PRP separation and >30 different types of commercially available PRP separation systems [130–132]. These different techniques yield PRP with platelet concentrations ranging from 0.52- to 9.3-fold relative to whole blood [133]. PRP differences are also caused by how and whether the platelets are activated before or when the PRP is applied, and the centrifugation parameters [132, 134, 135], which affect the ratios of unactivated vs. activated platelets, numbers of other different cell types, levels of bioactive factors, and leukocyte concentration [128, 130, 135–138], when platelets release their factors, (3) the ratios of the factors released, and (4) the level of factor bioactivity [139].

When comparing the analgesic efficacy of PRP, parameters that are never considered are how it is prepared and applied and the uncontrolled differences in the physiology, health, and products consumed by patients [128, 140, 141]. Thus, platelet aggregation, and therefore its efficacy, is increased by smoking [142], while platelet activation and aggregation are decreased by alcohol consumption [143], which also reduces the response of platelets to thrombin [144] and collagen. Platelet aggregation is also reduced by diets containing isoflavones [145], caffeine [146], quercetin (a flavonoid) [147], and anthocyanins [148]. However, platelet aggregation is increased by diets high in saturated fats [149], sugar [150], and simple carbohydrates [149]. Finally, platelets of patients with high blood pressure have a decreased whole blood platelet count [151], and their platelets have lower bioactive factor concentrations than those with normal blood pressure [152]. Therefore, without standardizing how PRP is prepared and applied, it is not possible to eliminate the variability in the efficacy of PRP and to ensure that PRP exerts its maximum potential effects [138].

### Novel clinical PRP application techniques induce long-term chronic neuropathic pain reduction/elimination

Recent case studies show that PRP induces long-lasting and complete pain elimination. These applications involved bridging nerve gaps with an autograft within a PRP-filled collagen tube [153–158] or only a PRP-filled collagen tube [159, 160]. The first technique reduced the pain in 8% and eliminated it in 92% of subjects, while the second eliminated pain in all the subjects. The pain reduction/elimination lasted throughout the 1.1–15 years follow-up. Thus, platelet-released factors have the capacity to induce long-term pain elimination.

### Novel PRP techniques are superior to TMR and RPNI

The novel PRP techniques are superior to TMR and RPNI. (1) They reduce/eliminate pain while promoting meaningful recovery. (2) While TMR is effective when applied up to 3 months post-trauma, its efficacy decreases with longer delays [72]. (3) RPNI requires survival or a small muscle graft, which PRP does not. (4) RPNI requires sacrificing a motor nerve, while using PRP does not.

### What underlies the high level of efficacy of the novel PRP application techniques?

While PRP provides short-term pain reduction/elimination [161, 162], the pain returns to 86% of patients [163]. This raises the question of why the novel PRP application techniques induce long-term pain reduction/elimination in 92%–100% of patients. The best explanation is in how the PRP was prepared and applied. First, applying PRP in a liquid form (without fibrin polymerization) causes the platelets to release all their factors within a few hours, while applying PRP in a polymerized fibrin form allows the platelets to release their factors over days [164], thus allowing the factors significantly more time to act. The novel PRP technique involved applying PRP in a polymerized fibrin form.

Second, although the optimal platelet concentration to provide maximal pain relief has not been determined, the degree of pain relief provided by PRP is reported to be linearly related to the number of platelets, the number and concentration of the growth and inflammatory factors they contain, and the number of neutrophils and monocytes [165]. Double centrifugation is the most commonly used PRP separation technique, yielding a ca. 4-fold increase in platelet concentration. This concentration is consistent with the finding that the degree of pain reduction associated with tennis elbow increases as the PRP platelet concentration is increased to 4-6-fold [166], while for knee osteoarthritis [167] and tendinopathies [168], a 3-4 fold concentration increase is recommended. However, it has also been reported that while a 2-fold increased platelet concentration yields good results for tissue healing, a 5-fold increase reduces healing [169, 170], and *in vitro* kills human tenocytes [171]. However, PRP used in the novel techniques prepared using GPS III concentrator tubes (Zimmer Biomet, Warsaw, IN) had a 9.3-fold increased platelet concentration.

Third, PRP from the GPS III concentrator tubes increased leukocytes by four-fold. Fourth, while most studies involve applying a small amount of PRP (≤1 mL), long-term pain relief is associated with the application of a significantly larger volume (4–6 mL). Fifth, long-term pain relief was associated with applying PRP to long (4–16 cm) vs. short (<0.5 cm) lengths of nerve [172]. Sixth, most studies showing a temporary analgesic influence of PRP involve applying it to the surface of nerves. However, PRP provides longer-lasting analgesia when the nerve and applied PRP are surrounded by a collagen tube. The tube reduces the diffusion of platelet-released factors away from the site, resulting in a higher effective concentration of the factors, which allows them to act on the axons for a longer time. This hypothesis is supported by the finding that the efficacy of PRP applied to a rat nerve crush site is increased by surrounding the application site with a collagen tube [112].

### Platelet-released factors

Platelets contain and release >300 identified factors [173]. While some are pre-packaged, with different types of platelet granules containing different factors, others are synthesized by platelets. The platelet’s environment determines the factor synthesis and release pattern [174–176]. Thus, physiologically, platelets release their factors in a specific order, with some released fast and others more slowly [173, 177, 178]. For example, nerve growth factor (NGF) is released within minutes, while brain-derived neurotrophic factor (BDNF) is released more slowly [176]. This sequence is critical to allow the factors to perform specifically timed functions, such as releasing pro-inflammatory factors first, followed by releasing anti-inflammatory factors, which suppress pain [179].

Platelet-released factors reduce/eliminate pain by additional mechanisms, but journal length limitations do not allow a complete discussion of the platelet-released factors that may be involved in reducing/eliminating pain. However, nerve injury induces voltage gated sodium channel (Na(v)) 1.3 channel expression in nociceptive and higher-order spinal sensory neurons, leading to their hyperexcitability, spontaneous ectopic electrical activity, and the development of neuropathic pain [180–182]. Therefore, one mechanism for reducing/eliminating chronic neuropathic pain is to down-regulate the expression of these channels, thus eliminating the spontaneous electrical activity that underlies pain.

Drugs, such as local anesthetics and other Na(v) channel-blocking techniques, reduce neuropathic pain by inhibiting nociceptive axon spontaneous ectopic nerve activity and hyperactivity [183–185]. The pharmacological blockade of sodium channel activity reduces ectopic electrical activity [185, 186] and reverses nerve injury-induced hyperalgesia [187]. This has been attributed to the blocking of Na(v) 1.8 and Na(v) 1.7 channels, leading to reduced or eliminated nociceptive neuron hyperexcitability. However, the role of Na(v)1.7 in neuropathic pain must be further investigated, and new analysis of a mouse Na(v)1.8 knockout suggests it is not involved in changing the neurons’ post-injury pain threshold following peripheral nerve injury [188]. However, it has also been shown that administering antisense oligonucleotides against Na(v)1.8 administered intrathecally completely reverses neuropathic pain behavior [189].

This is in contrast to the finding of Lai et al who reported that antisense oligonucleotides directed against Na(v)1.8 administered intrathecally completely reverse neuropathic pain behavior [18]. It is possible that this discrepancy could be due to the up-regulation of the Na(v)1.7 channel seen in the Na(v)1.8 knockout mouse [12], which might mask an otherwise important role for Na(v)1.8 in neuropathic pain.

However, the pain suppression is not long-lasting. However, in rats, nerve injury-induced chronic pain is reduced by Na(v)1.3 knockdown [190]. Further, intrathecal IL-10 infusion reduces neuropathic pain [191, 192] in part by down-regulating sodium channel expression in dorsal root ganglion (DRG) neurons [179], resulting in blocking nociceptive neurons’ hyperexcitability and spontaneous ectopic electrical activity [21–23, 185, 189, 193]. Although platelets do not contain IL-10, they release large amounts of prostaglandin E2 (PGE2), which induces interleukin-10 (IL-10) release [194, 195], which reduces pain [196–198]. Thus, multiple platelet-released factors can induce long-term chronic neuropathic pain reduction/elimination.

## Conclusion

Tissue injury-induced inflammation is the primary trigger of neuropathic pain, with chronic inflammation resulting in chronic pain. Injury induces the release of pro-inflammatory factors from local cells and other cells recruited to the injury site. While inflammation and pain are adverse events, they are required to trigger the normal physiological responses that induce the transition of a pro-inflammatory environment into an anti-inflammatory one, which is necessary for healing and pain elimination. Although some factors initially play pro-inflammatory roles, over time, they begin to play anti-inflammatory roles. Their roles depend on when they act after injury, what other factors are present, the cells on which they act, and the receptors on those cells. Thus, controlling pain requires controlling which factors are released and when. Platelets are an evolutionarily developed toolbox containing a physiological cocktail of factors for controlling cellular environments to promote healing and pain relief. While most studies find PRP only induces short-lived pain relief, two novel clinical techniques show that PRP can induce long-term chronic neuropathic pain elimination in all subjects. Further studies must determine which platelet-released factors, ratios, and concentrations induce these effects.
